# Deep Learning–Based Precision Cropping of Eye Regions in Strabismus Photographs: Algorithm Development and Validation Study for Workflow Optimization

**DOI:** 10.2196/74402

**Published:** 2025-07-17

**Authors:** Dawen Wu, Yanfei Li, Zeyi Yang, Teng Yin, Xiaohang Chen, Jingyu Liu, Wenyi Shang, Bin Xie, Guoyuan Yang, Haixian Zhang, Longqian Liu

**Affiliations:** 1 Department of Ophthalmology, West China Hospital, Sichuan University, 37 Guoxue Xiang (Alley), Chengdu, Sichuan Province, 610041, China, 86 18980601759; 2 Machine Intelligence Laboratory, College of Computer Science, Sichuan University, Chengdu, 610041, China

**Keywords:** artificial intelligence, ocular alignment, image preprocessing, clinical workflow, AI management system

## Abstract

**Background:**

Traditional ocular gaze photograph preprocessing, relying on manual cropping and head tilt correction, is time-consuming and inconsistent, limiting artificial intelligence (AI) model development and clinical application.

**Objective:**

This study aimed to address these challenges using an advanced preprocessing algorithm to enhance the accuracy, efficiency, and standardization of eye region cropping for clinical workflows and AI data preprocessing.

**Methods:**

This retrospective and prospective cross-sectional study utilized 5832 images from 648 inpatients and outpatients, capturing 3 gaze positions under diverse conditions, including obstructions and varying distances. The preprocessing algorithm, based on a rotating bounding box detection framework, was trained and evaluated using precision, recall, and mean average precision (mAP) at various intersections over union thresholds. A 5-fold cross-validation was performed on an inpatient dataset, with additional testing on an independent outpatient dataset and an external cross-population dataset of 500 images from the IMDB-WIKI collection, representing diverse ethnicities and ages. Expert validation confirmed alignment with clinical standards across 96 images (48 images from a Chinese dataset of patients with strabismus and 48 images from IMDB-WIKI). Gradient-weighted class activation mapping heatmaps were used to assess model interpretability. A control experiment with 5 optometry specialists compared manual and automated cropping efficiency. Downstream task validation involved preprocessing 1000 primary gaze photographs using the Dlib toolkit, faster region-based convolutional neural network (R-CNN; both without head tilt correction), and our model (with correction), evaluating the impact of head tilt correction via the vision transformer strabismus screening network through 5-fold cross-validation.

**Results:**

The model achieved exceptional performance across datasets: on the 5-fold cross-validation set, it recorded a mean precision of 1.000 (95% CI 1.000‐1.000), recall of 1.000 (95% CI 1.000‐1.000), mAP50 of 0.995 (95% CI 0.995‐0.995), and mAP95 of 0.893 (95% CI 0.870‐0.918); on the internal independent test set, precision and recall were 1.000, with mAP50 of 0.995 and mAP95 of 0.801; and on the external cross-population test set, precision and recall were 1.000, with mAP50 of 0.937 and mAP95 of 0.792. The control experiment reduced image preparation time from 10 hours for manual cropping of 900 photos to 30 seconds with the automated model. Downstream strabismus screening task validation showed our model (with head tilt correction) achieving an area under the curve of 0.917 (95% CI 0.901‐0.933), surpassing Dlib-toolkit and faster R-CNN (both without head tilt correction) with an area under the curve of 0.856 (*P*=.02) and 0.884 (*P*=.05), respectively. Heatmaps highlighted core ocular focus, aligning with head tilt directions.

**Conclusions:**

This study delivers an AI-driven platform featuring a preprocessing algorithm that automates eye region cropping, correcting head tilt variations to improve image quality for AI development and clinical use. Integrated with electronic archives and patient-physician interaction, it enhances workflow efficiency, ensures telemedicine privacy, and supports ophthalmological research and strabismus care.

## Introduction

Strabismus, marked by binocular misalignment, affects 0.8% to 6.8% [1] of children but can manifest at any age, impacting visual function, appearance, and social interactions including romantic relationships, learning, and employment opportunities [2]. Early detection and treatment are crucial, as they can considerably improve outcomes, underscoring the importance of timely intervention in managing this disorder. Recent initiatives have been directed towards crafting a dependable artificial intelligence (AI) system for diagnosing strabismus, utilizing ocular alignment photographs [3,4]. These images require isolation of the eye region to exclude identifiable facial features, ensuring privacy protection and preparing suitable datasets for further analysis. Historically, this process has relied on 2 manual and laborious tasks: cropping photographs and correcting for head tilts to achieve a horizontal alignment of the eyes [5,6]. This specificity in image preparation is not only critical for AI model training but also permeates clinical and academic settings, where there is a need to share or upload ocular alignment images that are devoid of any extraneous facial features. The manual nature of this conversion from full-face to eye-only images has highlighted the need for an automated, efficient solution to reduce the workload of medical professionals and health care costs.

Previous research has explored automating eye region cropping using algorithms like Dlib-toolkit [7,8] and faster region-based convolutional neural network (R-CNN) [9], which depend on facial landmarks and struggle with head tilt variations, often resulting in inconsistent cropping and loss of critical image features. This challenge is particularly significant in AI applications, where head tilt-induced aspect ratio inconsistencies, when resized to a uniform dimension, degrade model performance. Consequently, the automatically cropped photos often necessitate manual readjustment, limiting the practical application of the developed algorithms.

**Figure 1.**
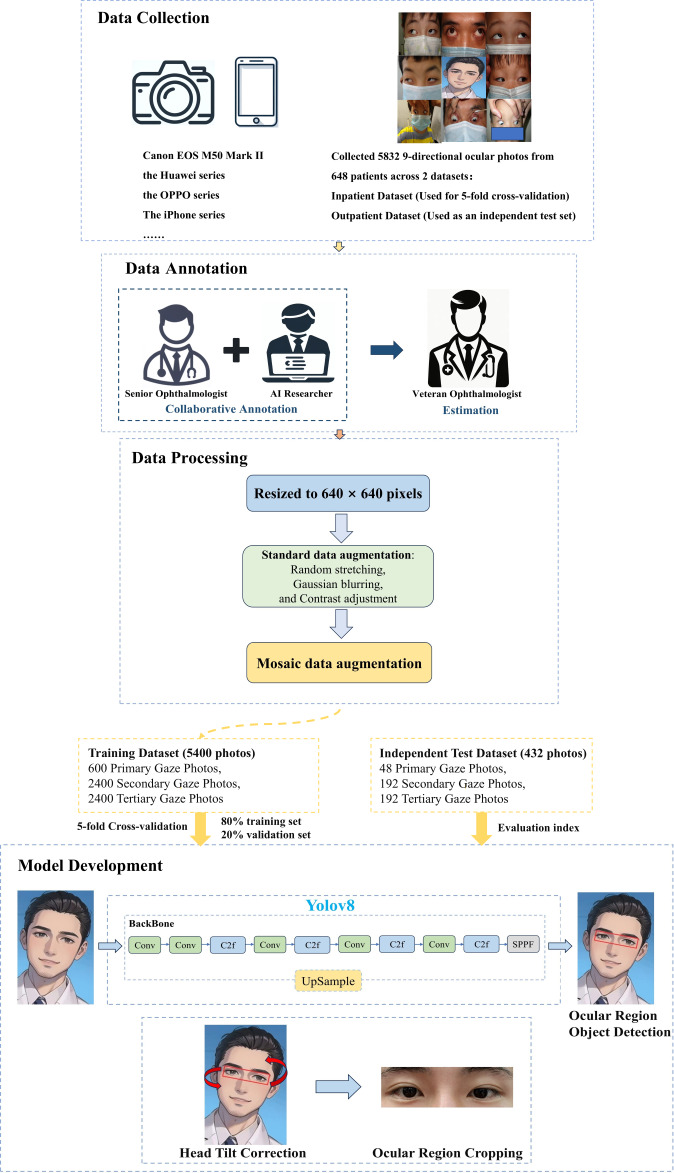
Workflow overview for intelligent eye region cropping in photographs of patients with strabismus. This workflow overview illustrates the comprehensive process undertaken in our study for intelligent eye region cropping in photographs of patients with strabismus. Beginning with data collection from 648 patients, we meticulously annotated eye regions, processed images for uniformity, and developed the model through systematic training and optimization strategies. Our approach encompasses both technical rigor in model architecture and practical considerations in data handling, ensuring precise eye region detection across diverse ocular positions. AI: artificial intelligence.

To address this gap, we developed an advanced rotating bounding box detection algorithm, built upon the YOLOv8 [10] backbone, to automatically crop the eye and periocular region while correcting for head tilt angles, as illustrated in Figure 1. Our approach was validated through a comparative evaluation of preprocessing methods using diverse datasets. Additionally, prior AI studies for strabismus have often focused on algorithm development, neglecting patient referral and long-term management, which can lead to gaps in care [6]. To address this, we developed an AI-driven management platform, available as a mobile applet for smartphones and tablets, integrating digital archives for patient test results and prescriptions, and a patient-physician interaction module to facilitate follow-up care, ensuring a seamless experience from initial assessment to ongoing monitoring.

This study aims to develop and validate an automated AI model for cropping the eye and periocular region in photographs of patients with strabismus, correcting for head tilt variations. By enhancing the efficiency, accuracy, and standardization of image preprocessing, the model optimizes clinical workflows, facilitating the construction of electronic health records. Additionally, the algorithm contributes to the development of high-quality, standardized datasets, thereby supporting subsequent downstream AI research.

## Methods

### Ethical Considerations

This study adhered to the ethical principles outlined in the Declaration of Helsinki and received approval from the Ethics Committee of West China Hospital, Sichuan University, China (2023 Review number 1477). Informed consent was obtained from all participants, and the research complied with local, national, regional, and international laws and regulations regarding the protection of personal information, privacy, and human rights. Additionally, no identifiable features of research participants are visible in any images included in the manuscript or supplementary materials.

### Data Collection

This retrospective and prospective cross-sectional study received approval from the Ethics Committee of West China Hospital, Sichuan University, China (2023 Review number 1477). Figure 1 delineates the study’s comprehensive workflow. Data were amassed from 3 distinct datasets to support model training, validation, and testing across diverse conditions. The training and validation set (5-fold cross-validation), derived from inpatients, included photographs of patients with strabismus across Primary Gaze, Secondary Gaze, and Tertiary Gaze positions, with each patient contributing 1, 4, and 4 photographs respectively, amounting to a total of 9 distinct eye positions, showcasing variability in distances. This dataset, consisting of 5400 ocular gaze photographs from 600 patients, was stratified and randomly split into training and validation sets following an 80% to 20% ratio for a 5-fold cross-validation process. These images were captured from patients both pre- and postsurgery, admitted to the Department of Ophthalmology at West China Hospital, China, from January 2018 to October 2023, using a Canon EOS M50 Mark II single-lens reflex autofocus digital camera, with a 24-million-pixel resolution and equipped with a lens-mounted flash. The camera was positioned at a distance ranging from 33 centimeters to 1 meter from the participants. The internal independent test set, comprising 432 photographs from 48 outpatients, was collected from August 1, 2023, to October 1, 2023, to evaluate the model’s performance under diverse imaging conditions. Photographs of 12 patients were captured using the EOS M50 Mark II (Canon Inc), while the remaining 36 patients’ photographs were taken with the Huawei (Huawei Inc), OPPO (OPPO Inc) series, and other leading mobile phone brands. The capturing devices were positioned at distances ranging from 33 centimeters to 1 meter from the patients.

To address concerns regarding the generalizability of the model across diverse populations and global clinical settings, an external cross-population test set was constructed using the IMDB-WIKI dataset [11], the largest publicly available face image dataset comprising 523,051 images. From this dataset, 500 full-face photographs were randomly selected, representing a wide range of age groups and ethnicities. This external test set was used to assess the model’s robustness and performance across varied facial features and skin tones, thereby enhancing the validation of its applicability in international contexts.

### Data Annotation

In this study, we used roLabelImg 3.0 software [12] for precise eye region annotation in photographs of patients with strabismus, a necessary step for developing our intelligent cropping algorithm. This tool allows for the detailed marking of rotated rectangle regions, crucial for accurately capturing the varied orientations of patients’ heads and eyes. A senior ophthalmologist and an AI medicine expert from the Computer Science department collaboratively annotated each image, with every annotated image subsequently reviewed by a Veteran Ophthalmologist boasting over 35 years of clinical and research experience. This ensured the identification of the eye region with high accuracy. The capability of roLabelImg to create adjusted bounding boxes for rotations was especially beneficial for our dataset, which includes photographs where faces and eyes often deviate from the horizontal. This process enabled precise annotations, accommodating head tilts and eyelid positions, which are typical in strabismus cases. These annotations are critical for training and testing the model, providing it with a comprehensive understanding of the eye region’s geometry, and significantly improving its performance in accurately cropping the eye region from full-face photographs.

### Data Processing

To maintain the original aspect ratio, all images were resized to 640×640 pixels via letterboxing. Moreover, beyond using standard data augmentation techniques such as random stretching, Gaussian blurring, and contrast adjustment, a crucial enhancement strategy was the implementation of Mosaic data augmentation. This technique amalgamates 4 distinct images—each from a different patient—into a single composite by blending their facial features, as illustrated in Figure S1 in Multimedia Appendix 1. By integrating the characteristics of 4 patients’ facial photographs, Mosaic augmentation enables the model to better discern the eye region as the primary focus, minimizing distractions from individual facial variations. This approach substantially broadens the training dataset’s variety, enhances the model’s generalization ability, and strengthens its capacity to interpret complex visual environments, thereby improving overall robustness and performance in diverse conditions.

### Model Development and Performance Evaluation

#### Model Architecture and Training Process

Considering the model’s speed and efficiency, we adopted YOLOv8 as the backbone. The backbone component incorporates the C2f module to improve gradient flow throughout the network, enabling certain upsampling operations to be executed with a single CNN layer and thus simplifying the network architecture. Network pruning was achieved via sparse training by selectively eliminating parameters based on their impact on model performance. For the head section, a decoupled head along with an anchor-free mechanism was implemented. This design segregates bounding box regression and classification into distinct pathways. Specifically, for this task, which requires only bounding box regression without the need for classification, such architecture minimizes the influence of classification on detection tasks, thereby boosting detection efficacy. The model was trained over 300 epochs, using a linear learning rate schedule with a warmup phase. Stochastic gradient descent served as the optimizer, with a batch size of 128 and an initial learning rate of 0.01. Furthermore, a foundational weight decay of 0.005 was applied during training. This comprehensive approach not only optimizes model performance but also enhances computational efficiency, supporting streamlined and effective object detection.

#### Optimization Strategy

Considering the characteristics of this task, we utilized only regression loss as the model’s total loss. The regression loss comprises distribution focal loss (DFL) and ProbIoU loss, with DFL_Reg_max set to 16 by default. ProbIoU harnesses the properties of rotated boxes, enhancing the conventional intersection over union (IOU) calculation by incorporating angle information, thereby facilitating the computation of rotated box IOU (refer to Section 4.3 for details). Unlike modeling a single Dirac distribution for bounding box coordinates, DFL loss regresses an arbitrary distribution to model the boundary box. Given the complexity of precisely defining the boundaries of eye sockets, slightly larger boxes are considered acceptable. DFL loss allows the network to focus promptly on values near the labels, maximizing probability density at the labels and thus constraining the model’s predictions within an acceptable range. This method uses the cross-entropy function to optimize probabilities around the label y, concentrating the network distribution near the label values. The final overall loss is derived by combining these 2 components with appropriate weighting.


$$B_{1}=\frac{1}{4}\frac{\left(a_{1}+a_{2}\right){\left(y_{1}-y_{2}\right)}^{2}+\left(b_{1}-b_{2}\right){\left(x_{1}-x_{2}\right)}^{2}+2\left(c_{1}+c_{2}\right)\left(x_{2}-x_{1}\right)\left(y_{1}-y_{2}\right)}{\left(a_{1}+a_{2}\right)\left(b_{1}+b_{2}\right)-{\left(c_{1}+c_{2}\right)}^{2}}$$

$$B_{2}=\frac{1}{2}ln\frac{\left(a_{1}+a_{2}\right)\left(b_{1}+b_{2}\right)+{\left(c_{1}+c_{2}\right)}^{2}}{4\sqrt{\left(a_{1}b_{1}-{c_{1}}^{2})((a_{2}b_{2}-{c_{2}}^{2}\right)}}$$

$$L_{Prob}\left(p,q\right)=1-ProbIoU\left(p,q\right)\;\in \left[0,1\right]$$

$$L_{DFL}\left(S_{i},S_{i+1}\right)=-\left(\left(y_{i+1}-y\right)\log\left(S_{i}\right)+\left(y-y_{i}\right)log\left(S_{i+1}\right)\right)$$

$$L_{total}={0.8*L}_{Prob}+{0.2*L}_{DFL}\left(S_{i},S_{i+1}\right)$$

#### IOU Calculation for Skewed Rectangles

**Figure 2.**
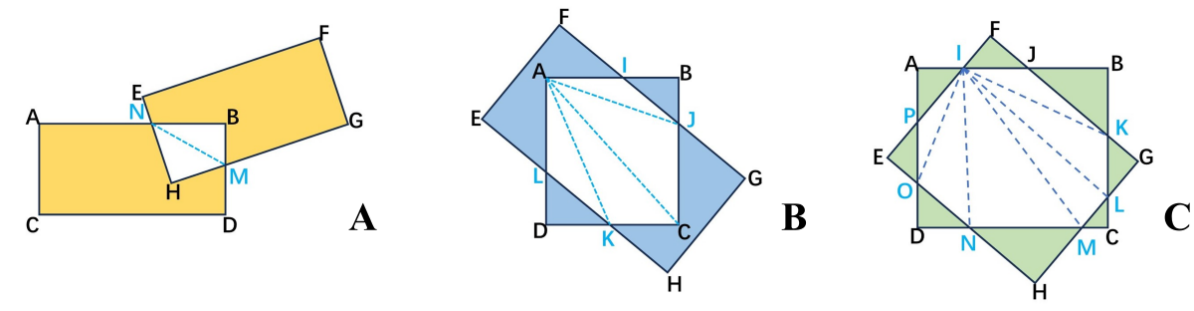
Geometric principles of skew intersection over union (IOU) calculation using triangulation. This figure depicts the process of skew IOU calculation through triangulation, illustrating the geometric principles involved. Rectangles ABCD (prediction) and EFGH (real) intersect to form set P with points I, J, K, and L, and vertices A and C. These points are sorted to create a convex polygon AIJCKL, which is then divided into triangles △AIJ, △AJC, △ACK, and △AKL for area calculation. This method enables precise IOU measurement by incorporating the angle information of skew rectangles.

We implement a method for calculating the skew IOU, incorporating triangulation. Figure 2 illustrates the geometric principles. Our objective is to calculate the IOU for each pair of skew rectangles. Consider rectangle ABCD as the prediction and rectangle EFGH as the artificially designed, real rectangle. First, we add the intersection points of the two rectangles to set P (eg, points I, J, K, and L in Figure 2B), including the vertices of one rectangle located within the other into set P (eg, points A and C in Figure 2B). Then, we sort the points in set P to form a convex polygon (eg, polygon AIJCKL in Figure 2B). Triangulation yields a set of triangles (shown in Figure 2B as △AIJ, △AJC, △ACK, △AKL). The polygon’s area is the sum of these triangles’ areas. Finally, this setup allows us to calculate the IOU using the intersection to union ratio formula:


$$IoU=\frac{Area\left(I\right)}{Area\left(rectangle_{ABCD}\right)+Area\left(rectangle_{EFGH}\right)-Area\left(I\right)}$$

#### Evaluation Metrics

The trained model’s performance is evaluated using precision rate, recall rate, and mean average precision (mAP) at various IOU thresholds. The precision rate measures the proportion of correctly predicted eye region boxes, while the recall rate evaluates how well the model identifies all relevant eye regions. The mAP50 assesses precision at a 50% IOU threshold, and mAP95 averages precision across IOU thresholds from 50% to 95%, offering a comprehensive measure of model accuracy [13]. The evaluation was conducted across 3 datasets: the 5-fold cross-validation set, comprising 5400 eye position photos, was used to ensure model robustness through cross-validation, with precision, recall, and mAP metrics computed for each fold; the internal independent test set, consisting of 432 eye position photos, served to validate performance on an independent dataset; and the external cross-population test set, derived from 500 randomly selected full-face photographs from the IMDB-WIKI dataset, assessed the model’s generalizability across diverse age groups, ethnicities, facial features, and skin tones. The final model was selected based on the best-performing fold from the cross-validation set and subsequently tested on both the internal independent test set and the external cross-population test set to evaluate its performance across the aforementioned metrics. Additionally, an expert in pediatric and strabismus ophthalmology with 35 years of clinical and research experience was consulted to evaluate whether the model’s eye region recognition and cropping performance meet clinical and research standards (Results section depicts actual images of eye region recognition and cropping by the model).

To further validate the model’s efficiency, a control experiment was conducted in which 5 optometry specialists each manually cropped and uploaded 81 eye position photos from 9 patients, with each patient contributing 1 photo in Primary Gaze, 4 in Secondary Gaze, and 4 in Tertiary Gaze positions. The average time per patient required by each physician to crop and upload these photos was recorded and compared with the average time taken by the model for the same task. This comparison aims to explore the model’s potential to reduce image processing time and assess its suitability for real-time clinical applications.

#### Model Interpretability Analysis

Grad-CAM (gradient-weighted class activation mapping) [14] was used to enhance the model’s interpretability. This technique was utilized to generate heatmaps, visualizing the regions of interest within full-face photographs.

#### Downstream Task Validation

To quantitatively evaluate the impact of preprocessing variations on downstream strabismus screening, we utilized our previously proposed state-of-the-art model (vision transformer [ViT]) [5] with 1000 first-gaze photographs. Three preprocessing approaches were compared: Dlib-toolkit and faster R-CNN without head tilt correction, and our developed model with automated head tilt correction. Each method was applied to preprocess the photographs, followed by 5-fold cross-validation training and evaluation using the ViT model. Performance was assessed through Accuracy, Precision, Specificity, Sensitivity, *F*_1_-score, and area under the curve (AUC) metrics, providing a numerical comparison of how head tilt correction influences model performance in a clinical screening context.

## Results

### Baseline Characteristics of the Study Participants

In this study, we harnessed a dataset from 648 patients with 5832 images across 3 gaze positions. The demographic and image attributes of participants within our study’s datasets are detailed in Table S1 in Multimedia Appendix 1, with the distributions of ocular alignment angle (Training set: median 0, range −12.603-5.737; Internal Testing set: median 0, range −5.713-5.737) and eye region bounding box area (Training set: median 0.134, range 0.022-0.305; Internal Testing set: median 0.127, range 0.024-0.249) depicted in Figure S2A,B in Multimedia Appendix 1. These 2 variables are pivotal in the clinical acquisition of ocular position photographs. The ocular alignment angle, measuring the angle (radians) between the line connecting the eyes’ inner canthi and the horizontal plane, is particularly significant due to the frequent occurrence of head tilt in patient images. This metric enables the model to autonomously correct for head tilt, ensuring the eyes are aligned horizontally. The eye region bounding box area, expressed as a percentage, reflects the eye region’s proportion of the entire image frame, highlighting the importance of accounting for nonuniform shooting distances and different device magnifications, which lead to bounding boxes of the eye region that vary significantly in size. This aspect of variability highlights our model’s adaptability and robustness, efficiently navigating the complexities introduced by diverse photographic conditions to achieve precise detection outcomes.

### Model Performance Evaluation

#### Quantitative Model Performance and Responsiveness

**Figure 3.**
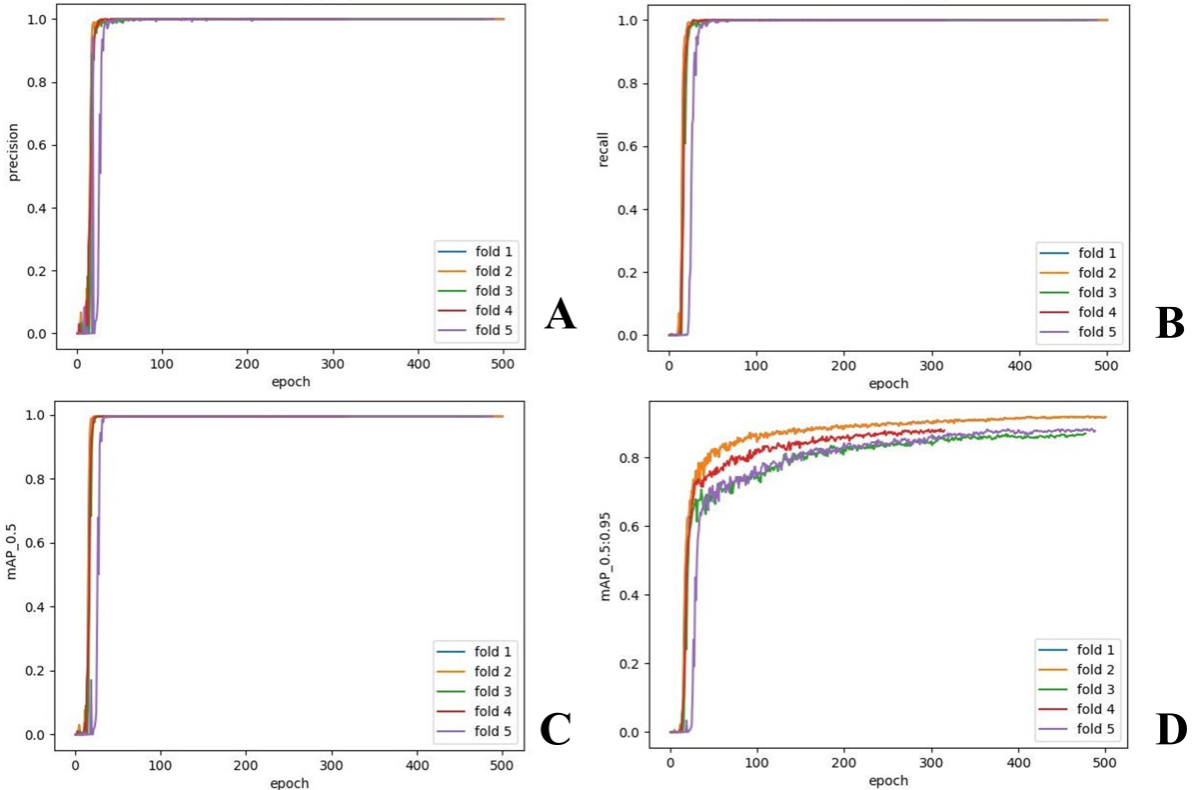
Performance curves on 5-fold cross-validation. This figure demonstrates the model’s performance across 5 validation folds during training. (A) Precision curves, all approaching 1.0, indicate high accuracy in eye region detection. (B) Recall curves, also nearing 1.0, reflecting robust identification of true positives. (C) mAP50, confirming the model’s precision across all folds. (D) mAP95, depicting consistent improvement at stricter intersections over union thresholds. mAP: mean average precision.

The model achieved a mean precision and recall of 1.000 (95% CI 1.000‐1.000), a mAP50 of 0.995 (95% CI 0.995‐0.995), and a mAP95 of 0.893 (95% CI 0.870‐0.918) across the 5-fold cross-validation set. The model’s steady convergence during training is visualized in Figure 3. On the internal independent test set, the model maintained a precision and recall of 1.000, with a mAP50 of 0.995 and a mAP95 of 0.801. Additionally, on the external cross-population test set, the model exhibited a precision and recall of 1.000, a mAP50 of 0.937, and a mAP95 of 0.792, further validating its performance across diverse populations, as presented in Table 1. Manually cropping and uploading 9 photos per patient took specialists an average of 6.52 minutes, with a near-normal time distribution across physicians (Figure S3 in Multimedia Appendix 1). By contrast, the model completed the task in just 27 milliseconds per photo, totaling 0.243 seconds for all 9 photos, underscoring its efficiency for real-time clinical use.

**Table 1.** Model performance across validation and test sets.

Dataset	Precision	Recall	mAP50^a^	mAP95^b^
5-fold cross-validation set, mean (95% CI)	1.000 (1.000-1.000)	1.000 (1.000-1.000)	0.995 (0.995-0.995)	0.893 (0.870-0.918)
Internal independent test set	1.000	1.000	0.995	0.801
External cross-population test set	1.000	1.000	0.937	0.792

a mAP50: mean average precision at 50% intersection over union threshold.

b mAP95: mean average precision averaged over intersection over union thresholds from 0.5 to 0.95.

#### Qualitative Expert Validation

**Figure 4.**
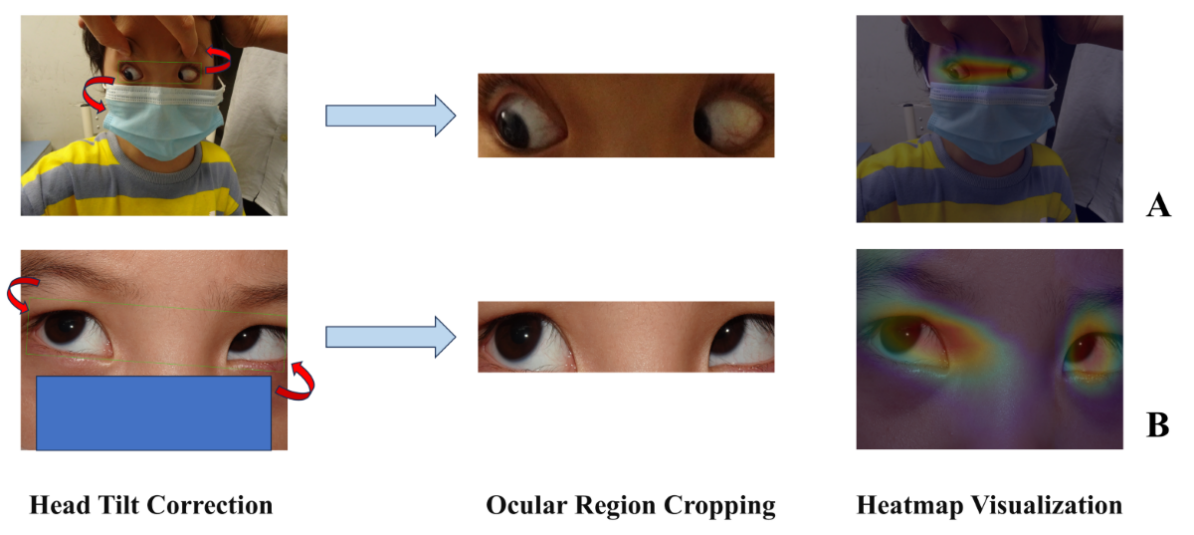
Head tilt correction, ocular region cropping, and heatmap visualization in images of patients with strabismus. This figure illustrates the model’s processes across 2 photographs of patients with strabismus, with gradient-weighted class activation mapping heatmaps revealing that detection, cropping, and head tilt correction are primarily achieved by focusing on the core ocular regions of full-face images. (A) The ocular alignment angle is 0.457° and the eye region bounding box area is 0.054. (B) The ocular alignment angle is 2.382° and the eye region bounding box area is 0.171. The model isolates the eye region, adjusts image orientation for horizontal alignment, and offers user-adjustable cropping boundaries to accommodate varying clinical needs and enhance flexibility.

The model’s eye region detection and cropping were qualitatively assessed by a pediatric and strabismus ophthalmology expert with over 35 years of experience, assessing 96 images comprising 48 images of randomly selected photographs from a Chinese dataset of patients with strabismus and 48 images from the IMDB-WIKI dataset, as depicted in Figure S4 in Multimedia Appendix 1. These images span diverse ages, skin tones, ethnicities, and distances, including obstructions such as fingers and cotton swabs. The expert review affirmed the model’s precision and high-quality outcomes across these 2 varied datasets. Figure 4 showcases examples of head tilt correction and ocular region cropping for 2 patients. Such detailed evaluation confirms the model’s adherence to clinical and research standards, showcasing its capability in accurate eye region recognition and cropping.

#### Interpretability Analysis Using Grad-CAM Heatmaps

Building on the expert validation, Grad-CAM heatmaps were used to further elucidate the model’s decision-making transparency, as depicted in Figure 4. The heatmaps in Figure 4A and 4B reveal that the model predominantly concentrates on the core ocular regions of the full-face images, with warmer colors indicating higher attention to these areas. The highlighted regions’ tilt direction aligns with the patient’s head tilt, demonstrating that the model primarily performs target detection by focusing on the core ocular regions and accurately learns the head tilt angle information.

#### Impact of Cropping Methods on Downstream Strabismus Screening

The strabismus screening model (ViT) was evaluated using 1000 primary gaze photographs preprocessed with 3 cropping methods: Dlib-toolkit and faster R-CNN (both without head tilt correction) and our model (with head tilt correction). As shown in Table 2, our model outperformed the others, achieving an AUC of 0.917 (95% CI 0.901‐0.933), compared with 0.856 (95% CI 0.837‐0.875, *P*=.02) for Dlib-toolkit and 0.884 (95% CI 0.866‐0.902, *P*=.05) for faster R-CNN.

**Table 2.** Comparison of cropping methods on strabismus screening model (vision transformer [ViT]) performance. This table presents the performance of the strabismus screening model (ViT) based on 1000 primary gaze photographs, cropped using Dlib-toolkit, faster region-based convolutional neural network (R-CNN; both uncorrected for head tilt), and our model (corrected for head tilt), evaluated via 5-fold cross-validation. The *P* values, calculated using a paired *t* test, compare the area under the curve (AUC) of each method with that of our model, with *P*<.05 indicating statistical significance.

Cropping method	Accuracy, mean (95% CI)	Precision, mean (95% CI)	Specificity, mean (95% CI)	Sensitivity, mean (95% CI)	*F*_1_-score, mean (95% CI)	AUC, mean (95% CI)	*P* value (AUC vs our model)
Dlib-toolkit (uncorrected)	0.872 (0.853-0.891)	0.849 (0.829-0.869)	0.863 (0.842-0.884)	0.834 (0.811-0.857)	0.841 (0.820-0.862)	0.856 (0.837-0.875)	.02
Faster R-CNN (uncorrected)	0.896 (0.879-0.913)	0.875 (0.857-0.893)	0.882 (0.863-0.901)	0.861 (0.839-0.883)	0.868 (0.849-0.887)	0.884 (0.866-0.902)	.046
Our model (corrected)	0.921 (0.905-0.937)	0.904 (0.887-0.921)	0.912 (0.895-0.929)	0.895 (0.877-0.913)	0.899 (0.882-0.916)	0.917 (0.901-0.933)	—^a^

a Not available.

### AI-Driven Management Platform

**Figure 5.**
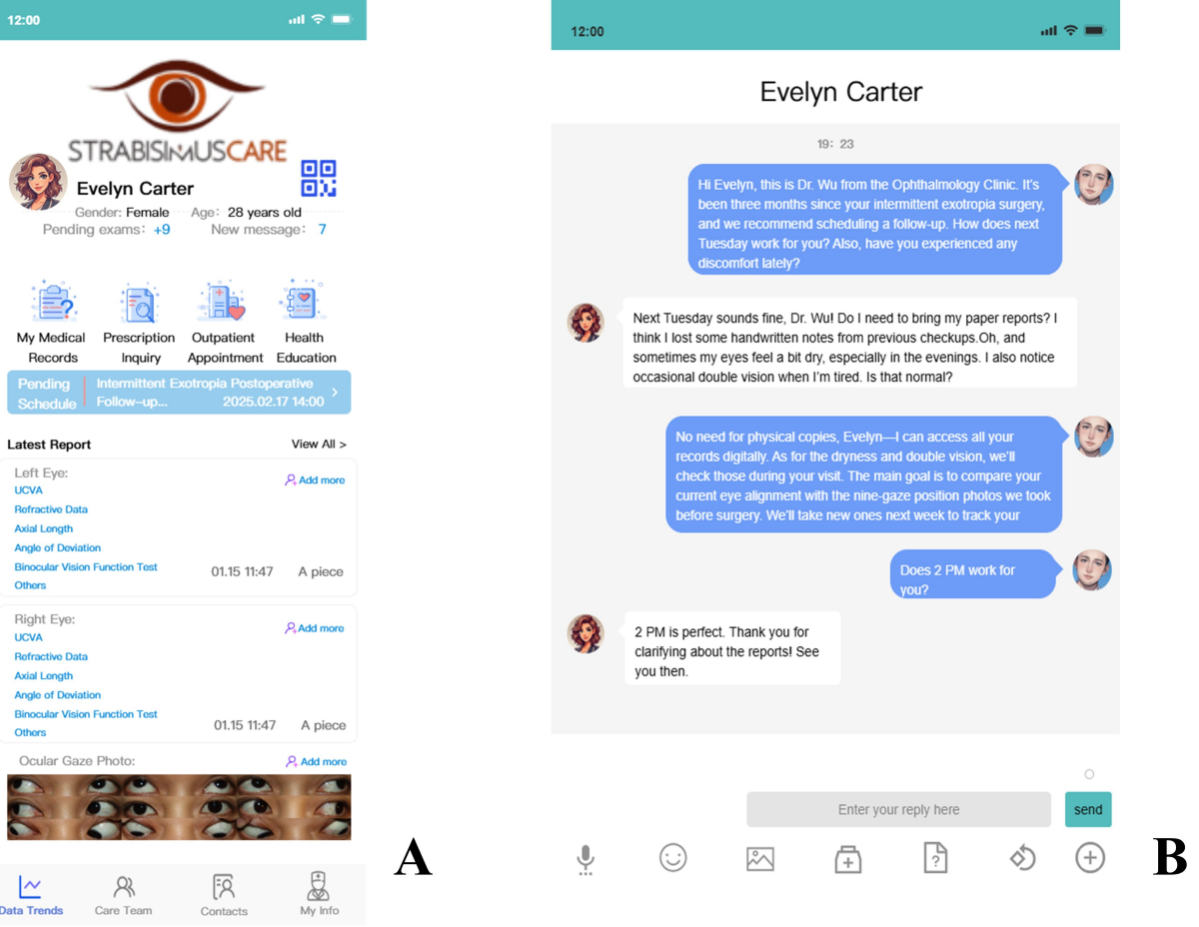
The interface of the artificial intelligence-driven management platform for the care of patients with strabismus. (A) Depicts the digital medical records interface, where patients and health care providers can access and manage comprehensive patient histories, including diagnoses, prescriptions, and eye examination results. (B) Depicts the doctor-patient interaction module, allowing seamless communication through text, images, and video, facilitating effective consultation and follow-up care.

The AI-driven management platform developed in this study not only enhances the precision of strabismus eye region cropping but also integrates functionalities that improve overall patient care (as shown in Figure 5).

#### Patient 9-Position Ocular Gaze Upload and Information Collection

The platform collects 9-position ocular gaze photographs. These photographs are captured by an ophthalmologist in a clinical setting, ensuring the accuracy and consistency of the data. To address potential failures or low-confidence scenarios, the system incorporates a dual-layer quality assurance process: premodel image quality checks and postmodel detection validation. Uploaded photos must have a minimum resolution of 5 megapixels. If this requirement is not met, the system immediately alerts the user to the inadequate resolution and requests a replacement image. Furthermore, if the detection algorithm finds that the eye region constitutes less than 2.5% or more than 25% of the total photo area—suggesting that the shooting distance deviates from the ideal range of 33 cm to 1 m—the system informs the user that the distance is either “too far” or “too close,” respectively, and prompts a repositioning adjustment. Post detection, if the model’s confidence score, reflecting its certainty in identifying the eye region, falls below 50%, the image is flagged as unreliable, potentially due to occlusion, motion blur, or poor lighting conditions. Users are then instructed to retake and upload a clearer image to ensure accurate detection. In addition, essential patient data, including name, gender, date of birth, UCVA, refractive data, axial length, angle of deviation, and binocular vision function test results—previously recorded on paper—are digitally stored. This process is guided and assisted by the attending physician, ensuring that all examination results are accurately uploaded, thus creating a comprehensive patient profile.

#### Digital Medical Records

A secure digital record system allows for easy access to patients’ diagnostic results and prescriptions. Both patients and health care providers can retrieve and review these records, ensuring that clinical decisions are based on up-to-date information. This feature also supports the integration of historical data, promoting continuity of care and aiding in both treatment progress tracking and informed clinical decision-making.

#### Patient-Physician Interaction Module

The platform includes an interactive module that allows seamless communication between patients and health care providers. This module facilitates consultation, sharing of symptoms, and response to inquiries, reducing the likelihood of missed follow-ups. Communication through text, images, and videos further improves the consultation process, providing personalized and efficient care.

## Discussion

This study developed and validated an automated AI model for cropping the eye and periocular region in photographs of patients with strabismus, demonstrating its efficacy across diverse datasets. The model achieved near-perfect precision and recall (1.000) on both the 5-fold cross-validation set (mAP50: 0.995, mAP95: 0.893) and internal independent test set (mAP50: 0.995, mAP95: 0.801), with robust performance on the external cross-population test set (mAP50: 0.937, mAP95: 0.792), confirming its generalizability across varied ethnicities and ages. Expert validation on 96 diverse images affirmed its precision, while Grad-CAM heatmaps revealed focused attention on core ocular regions, aligning with head tilt angles. Downstream evaluation using the ViT model showed superior performance with head tilt correction (AUC 0.917) compared with Dlib-toolkit (AUC 0.856) and faster R-CNN (AUC 0.884), highlighting the model’s clinical efficiency by reducing cropping time from 6.52 minutes to 0.243 seconds per patient.

Strabismus screening, traditionally manual and reliant on physician expertise, demands significant patient-doctor cooperation, often resulting in subjective outcomes. Recent efforts focus on developing AI systems using photographs for quicker, more objective diagnoses [5,6]. However, the advancement of strabismus screening AI has been significantly impeded by the laborious and prone-to-error task of manually cropping photographs to determine ocular alignment, particularly when correcting for head tilts in full-face images to achieve horizontal eye alignment. In clinical environments, such as outpatient clinics, there exists a parallel demand for an algorithm that enables clinicians to rapidly crop full-face photographs to isolate the eye region for inclusion in electronic health records, thereby preserving patient privacy and enhancing the quality of diagnostic imaging. This requirement is echoed in academic and telemedicine contexts, where the accuracy and precise alignment of eye region images are paramount. It is crucial to address these longstanding challenges by leveraging advanced technological solutions.

**Figure 6.**
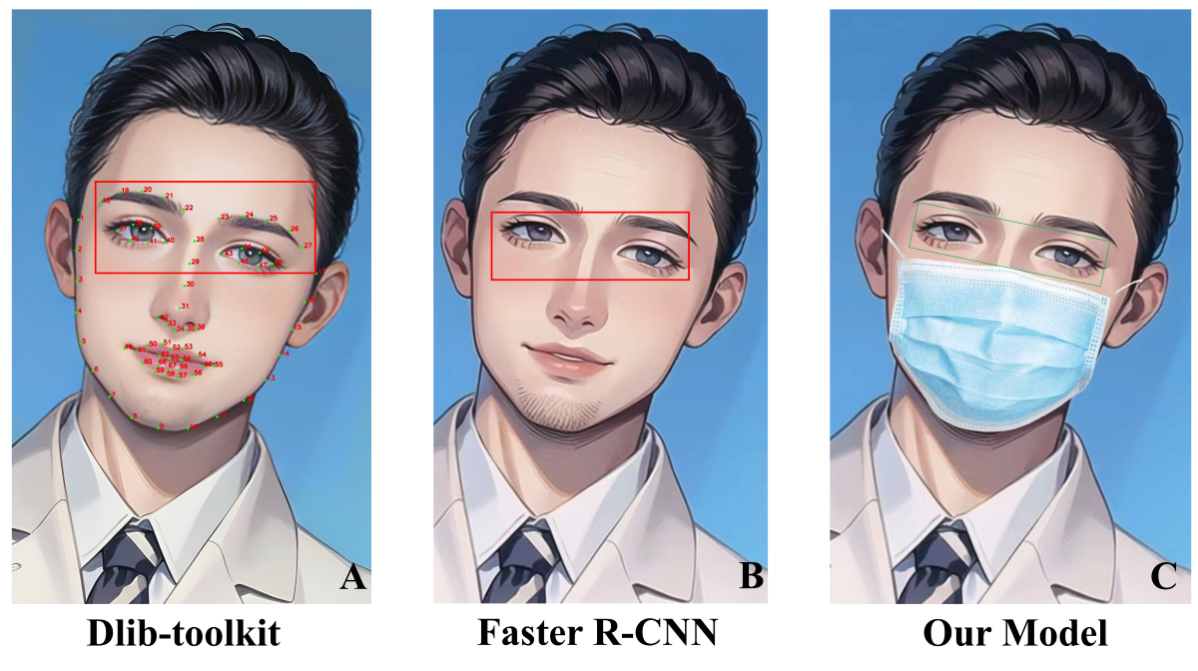
Comparative evaluation of eye region cropping algorithms from full-face patient photographs. This compares eye region cropping algorithms applied to cartoon-style full-face photographs, derived from the author’s own images, to reflect the universal applicability of these methods—even for cartoon characters. (A) The eye region cropping using the Dlib-toolkit facial feature point recognition function, which extracts 68 facial landmarks to identify and crop the eye region. The effectiveness of this method is contingent on the visibility of facial landmarks, which can be hindered by obstructions such as masks, fingers, or cotton swabs during eyelid retraction. (B) The application of the faster R-CNN object detection algorithm for eye region cropping. This method generates region proposals followed by classification and refinement, but it can be challenged by the misalignment of eyes and the variability in the aspect ratio of cropped images, often necessitating manual adjustments post cropping. (C) The results of our proprietary algorithm for eye region cropping are specifically designed to address head tilts and ensure horizontal alignment of the eyes. This innovative approach automates the cropping process while maintaining the original features of the eye region, reflecting significant improvements over prior methods. R-CNN: region-based convolutional neural network.

The previously proposed automatic eye region cropping algorithm was primarily based on the Dlib-toolkit facial feature point recognition function [7,8,15]. This function utilizes a facial feature point detector to extract 68 facial landmarks (as shown in Figure 6A) and locates the eye region based on the detected facial positional information for cropping. However, this method relies on additional facial landmarks to identify eye region markers, which becomes problematic in clinical settings where patients often wear masks, only the upper half of the face is photographed for clarity, or fingers and cotton swabs obstruct the extraction of facial landmarks during eyelid retraction. For such photos, the eye region cropping algorithm developed with the Dlib-toolkit facial feature point recognition function fails to operate. Furthermore, as the Dlib-toolkit function is encapsulated within the Pytorch platform, it cannot be modified.

Using object detection algorithms presents an alternative approach for cropping the eye region in full-face photographs. Presently, object detection algorithms are divided into 2 principal methodologies: two-stage and one-stage detection. Two-stage detectors, such as faster R-CNN [9], R-FCN [16], and Mask R-CNN [17], initiate by generating a multitude of region proposals within images, which are then classified and refined. This sequential process underpins the nomenclature of two-stage detection. There have been attempts to automate eye region cropping using the faster R-CNN object detection algorithm [18], with the results shown in Figure 6.B. However, this algorithm faces challenges such as misalignment of the eyes on the same horizontal plane affecting diagnostic accuracy and variation in the aspect ratio of cropped images due to patient head tilt, resulting in loss of original features when resized for model input. Consequently, images often require manual readjustment following automated cropping by the algorithm to rectify these discrepancies.

One-stage detectors, such as YOLO [19], SSD [20], and M2Det [21], streamline object detection by directly predicting categories and locations in a single step, bypassing proposal box generation. This efficiency renders them faster and more suitable for real-time tasks with limited computational resources than their two-stage counterparts. Within the array of one-stage detectors, the YOLO architecture distinguishes itself by striking an optimal balance between speed and precision, facilitating swift and dependable object recognition within images. Specifically, in medical applications, YOLO’s application spans cancer detection [22], skin lesion segmentation [23], and pill identification [24], contributing to heightened diagnostic accuracy and streamlined treatment protocols [13].

Our study developed a novel ocular gaze photograph preprocessing algorithm tailored for automated cropping of the eye and periocular region in full-face photographs of patients with strabismus. This algorithm effectively accounts for head tilt angles by initially cropping the full-face image to isolate the eye region and then realigning it to ensure horizontal eye alignment, handling tilt angles up to 5.74 radians, enhancing its robustness and utility in clinical and telemedicine settings where precise eye region identification is essential. The model demonstrated outstanding performance across 3 datasets: on the 5-fold cross-validation set, it achieved a precision and recall of 1.000, with a mAP50 of 0.995 and mAP95 of 0.893; on the internal independent test set, it maintained a precision and recall of 1.000, with a mAP50 of 0.995 and mAP95 of 0.801; and on the external cross-population test set, it exhibited a precision and recall of 1.000, a mAP50 of 0.937, and a mAP95 of 0.792, confirming its generalizability across diverse ethnicities and ages. Additionally, Grad-CAM heatmaps revealed that the model primarily focuses on the core ocular regions in full-face images, with the highlighted regions’ tilt direction aligning with the patient’s head tilt, indicating that the model performs target detection by concentrating on these areas and accurately learns head tilt angle information, enhancing its decision-making transparency. In a clinical setting, manually cropping and adjusting 900 eye position photos from 100 patients would take a physician approximately 10 hours—a process prone to human error and inconsistencies in aspect ratios. Our model completes this task in under 30 seconds, significantly improving the accuracy and efficiency of image preparation for downstream strabismus-related applications. Furthermore, prior research noted that head tilt variations result in inconsistent aspect ratios in cropped images, which, when resized to a uniform dimension for model input, lead to the loss of original image features and degraded performance [5,6]. Despite these challenges, earlier studies lacked quantitative evidence on the impact of head tilt correction. To address this gap, we conducted downstream task validation by preprocessing 1000 primary gaze photographs with 3 methods—Dlib-toolkit and faster R-CNN (both without head tilt correction) and our model (with head tilt correction)—and evaluated them using the ViT model through 5-fold cross-validation. Our model achieved an AUC of 0.917 (95% CI 0.901‐0.933), outperforming Dlib-toolkit (AUC 0.856, 95% CI 0.837‐0.875, *P*=.02) and faster R-CNN (AUC 0.884, 95% CI 0.866‐0.902, *P*=.05), with *P* values (<.05) confirming statistical significance. This improvement stems from head tilt correction, which mitigates the issue of inconsistent aspect ratios caused by varying head tilt angles, preserving original image features during resizing for model input and thereby enhancing the ViT model’s performance in strabismus-related applications. Additionally, the model offers a user-adjustable cropping boundary, allowing the inclusion of surrounding areas based on user preference, thereby enhancing its flexibility to meet diverse clinical needs.

Currently, many clinical records for patients with strabismus are still maintained in paper format, leading to issues with the nondigital and unstructured collection and annotation of data. These challenges result in incomplete or inaccurate multimodal medical data, which limits the broader application and development of AI research in this field [3,6]. Digital products in strabismus care remain scarce, and previous AI-driven screening and diagnostic efforts have typically only focused on delivering diagnostic results, lacking support for patient referrals and long-term follow-up. This gap often leads to missed follow-up visits, hindering comprehensive treatment and obstructing the collection of diverse, multimodal, and longitudinal data from patients [6]. Our platform integrates electronic archives to create a secure and efficient system for storing patient test results and prescriptions. This integration allows for comprehensive and accurate tracking of patient histories, enabling informed clinical decision-making. Additionally, the platform enhances patient management by preventing missed appointments and ensuring timely treatment. By leveraging advanced data management tools, it facilitates large-scale population data collection, which in turn supports improved intervention strategies and prognosis predictions. This innovation represents a major step forward in strabismus management, with the potential to significantly improve patient outcomes and optimize the delivery of eye care services. Given the absence of similar intelligent products previously, this study introduces an AI platform framework, which still lacks formal usability testing, user feedback, or satisfaction assessments, and awaits future real-world multicenter studies to explore its usability and health economic benefits for patient management.

In summary, this study presents a novel approach in ophthalmology, developing an advanced ocular gaze photograph preprocessing algorithm for precise eye region cropping in patients with strabismus. By automating a traditionally manual and error-prone process, our method significantly improves the accuracy and efficiency of image preparation for downstream applications and AI model development, thereby enhancing clinical workflows. Additionally, we developed a comprehensive AI-driven management platform, which integrates digital archives and patient-physician interaction modules, further optimizing patient care from initial assessment through to long-term follow-up.

## Supplementary material

10.2196/74402 Multimedia Appendix 1 Figures and a table supporting the study’s methodology and findings.

## Abbreviations

AI: artificial intelligence
AUC: area under the curve
DFL: distribution focal loss
ERBBA: eye region bounding box area
Grad-CAM: gradient-weighted class activation mapping
IOU: intersection over union
mAP: mean average precision
R-CNN: region-based convolutional neural network
ViT: vision transformer
