# Supplementary material for: Deep Learning–Based Precision Cropping of Eye Regions in Strabismus Photographs: Algorithm Development and Validation Study for Workflow Optimization
Source: J Med Internet Res. 2025 Jul 17;27:e74402. doi: 10.2196/74402 (PMC12293638; doi:10.2196/74402)
Supplement: Multimedia Appendix 1 [file jmir-v27-e74402-s001.docx]

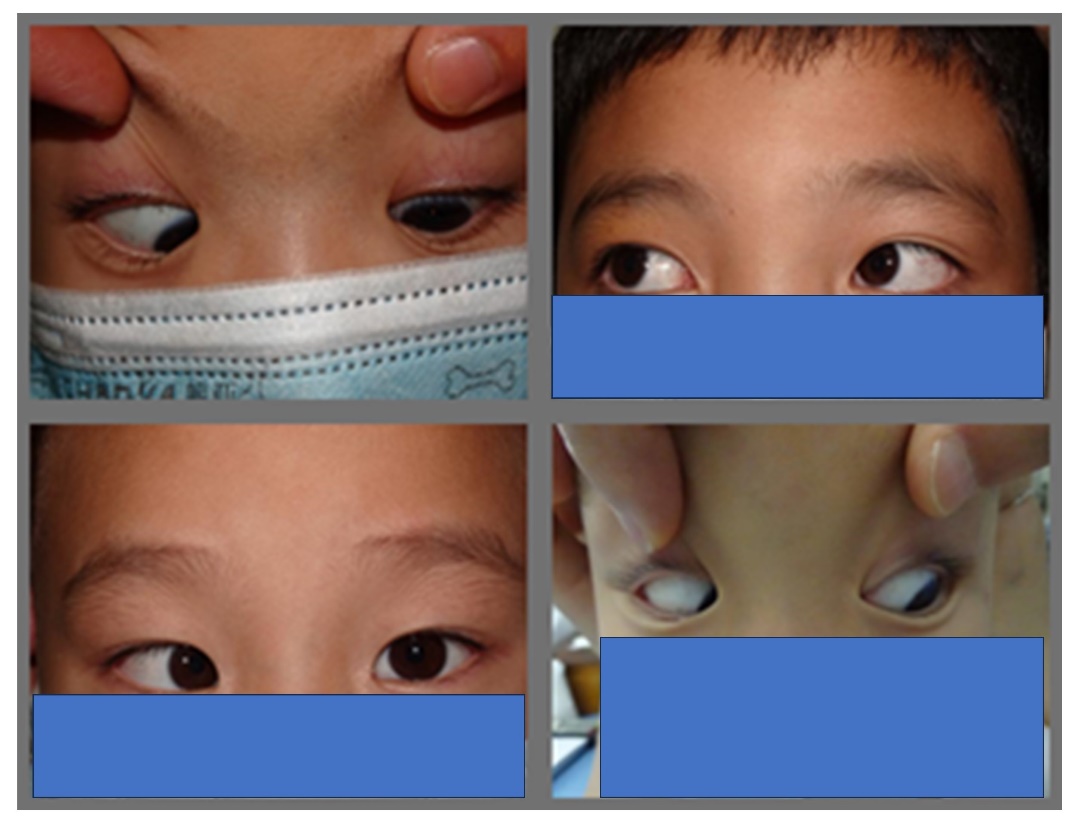


**Figure S1 Mosaic Data Augmentation: Enhancing Model Diversity with Composite Images**

Figure S1 illustrates the Mosaic data augmentation technique, where four distinct patient photographs are combined into a single composite image. This innovative approach enriches the training dataset by introducing a higher level of visual diversity, which in turn enhances the model's ability to understand and analyze complex visual scenarios. By integrating varied ocular positions and conditions within one image, the model learns to detect and interpret the eye region with greater accuracy and adaptability, significantly boosting its robustness and performance across different situations.


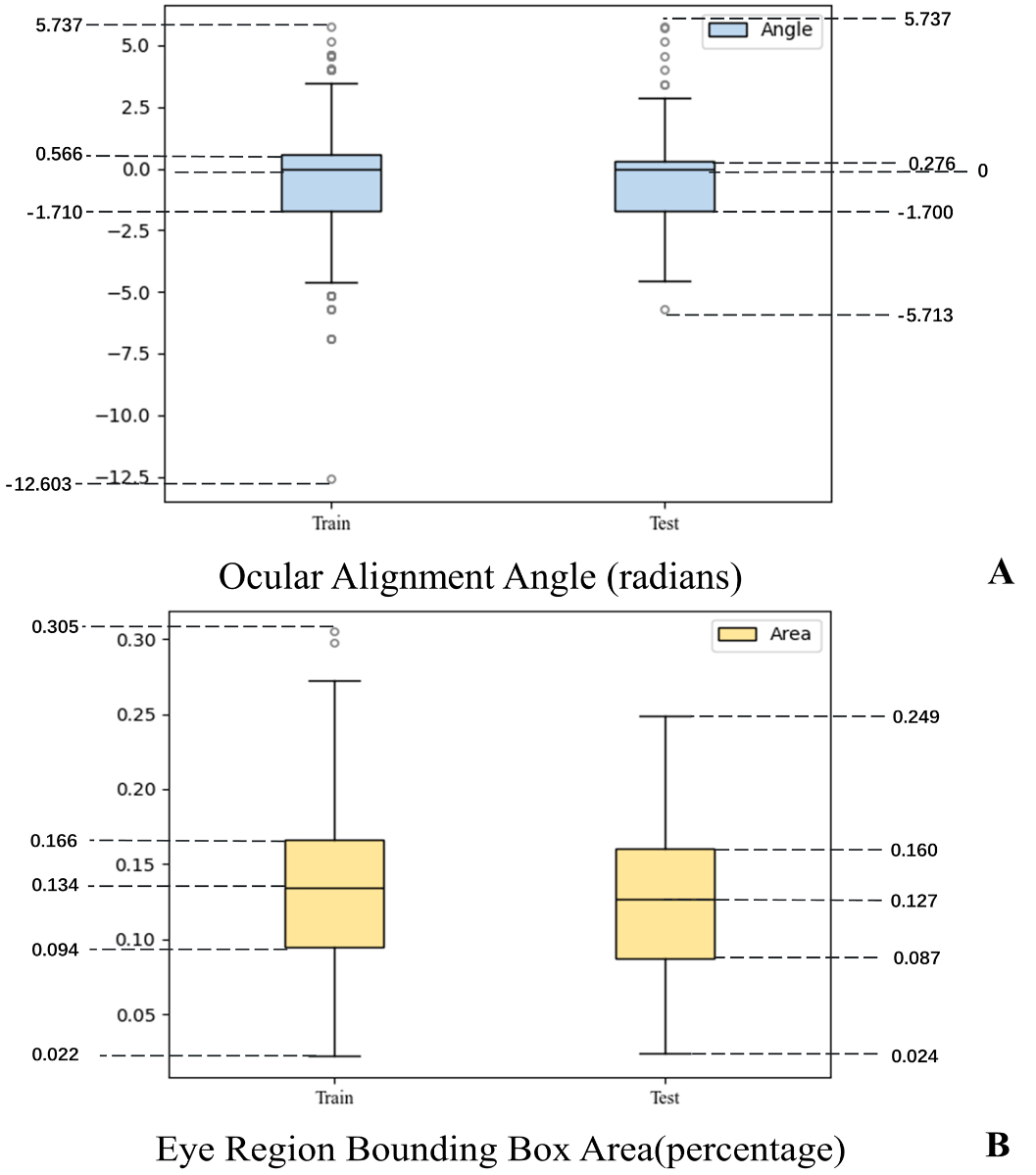


**Figure S2 Distribution of Ocular Alignment Angle and Eye Region Bounding Box Area in Strabismus Patient Photographs**

Figure S2.A illustrates the distribution of Ocular Alignment Angle (OAA) among the study participants. The OAA is critical for assessing the degree of head tilt in patient images, facilitating the model's capability to autonomously adjust and ensure horizontal alignment of the eyes.

Figure S2.B depicts the distribution of the Eye Region Bounding Box Area (ERBBA), calculated as the percentage of the eye region relative to the entire image frame. It illustrates the model's adeptness in handling the size variability due to different shooting distances and device magnifications, maintaining precise eye region cropping under diverse conditions.

**
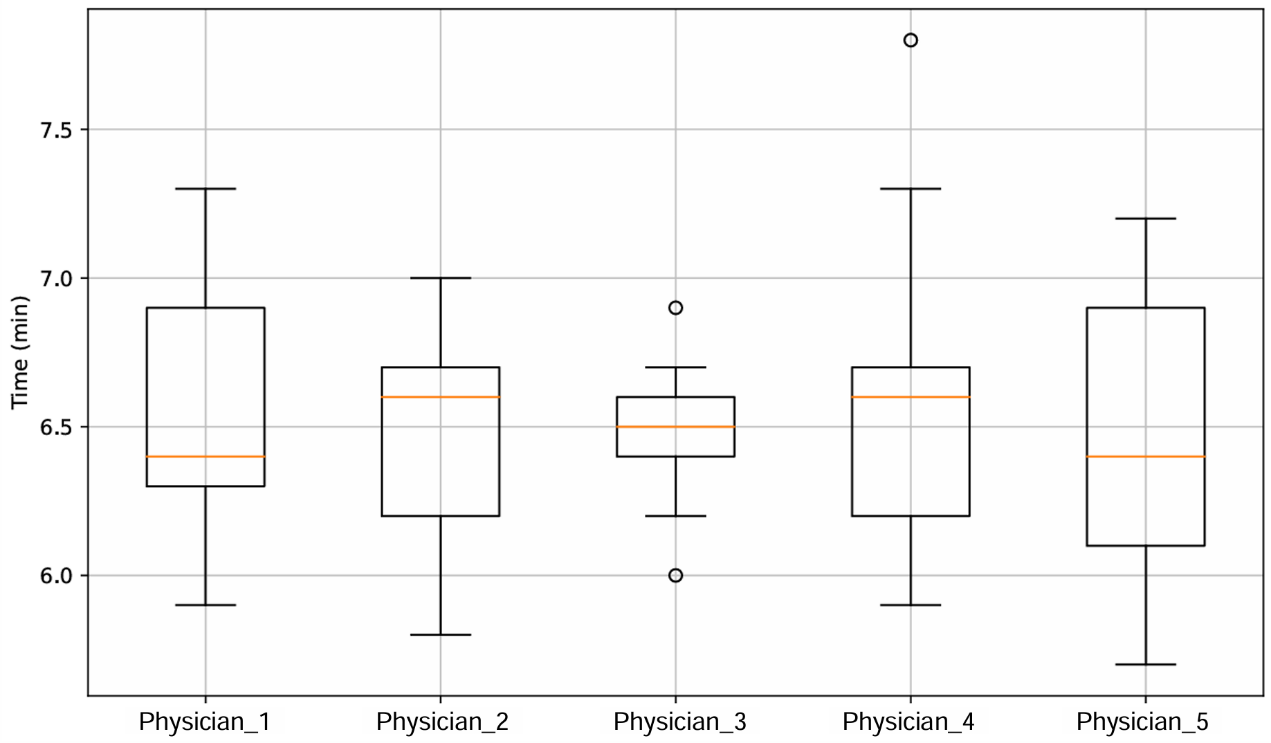
**

**Figure S3 Distribution of Time Required by Optometry Specialists for Patient Eye Position Image Processing**

Figure S3 illustrates the distribution of time required by five optometry specialists to process eye position images for nine patients. The median processing time for each physician is as follows: Physician 1 has a median of 6.4 minutes, Physician 2 has a median of 6.6 minutes, Physician 3 has a median of 6.5 minutes, Physician 4 has a median of 6.6 minutes, and Physician 5 has a median of 6.4 minutes. The interquartile ranges (IQR) are 1.3 minutes for Physician 1, 0.6 minutes for Physician 2, 0.5 minutes for Physician 3, 1.1 minutes for Physician 4, and 0.8 minutes for Physician 5. The whiskers extend to minimum and maximum times of 5.9 and 7.3 minutes for Physician 1, 5.8 and 7.0 minutes for Physician 2, 6.0 and 6.9 minutes for Physician 3, 5.9 and 7.3 minutes for Physician 4, and 5.7 and 7.2 minutes for Physician 5. Outliers are minimal, with only Physician 4 showing a slightly larger upper range of processing time.


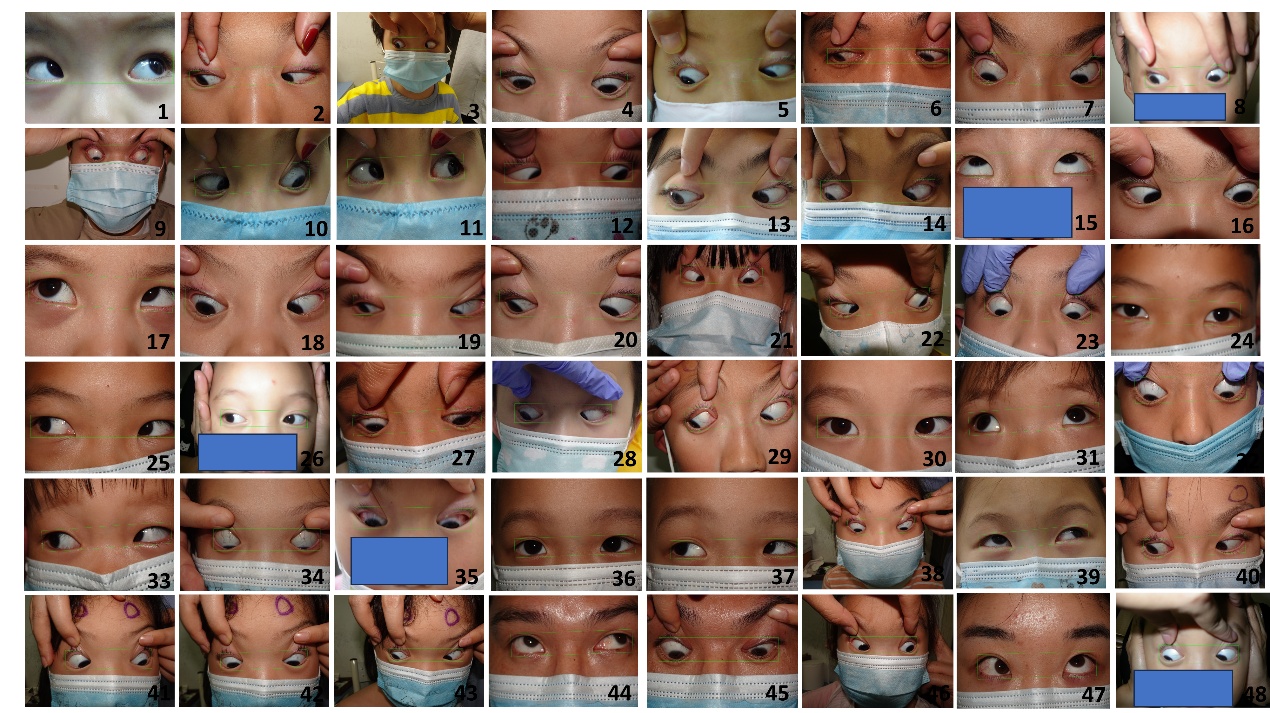


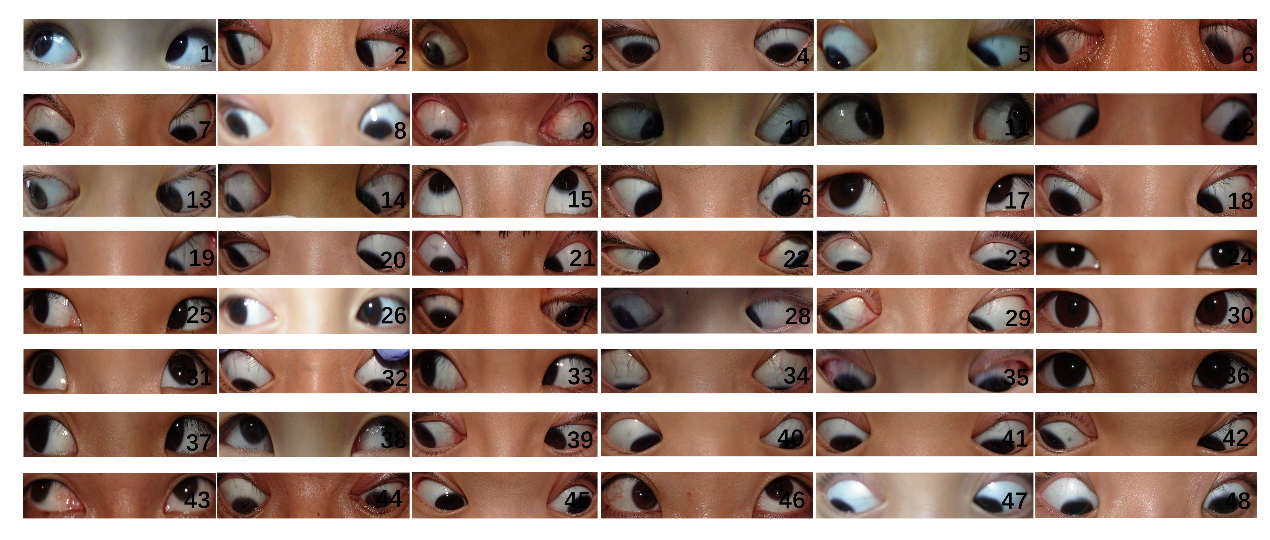


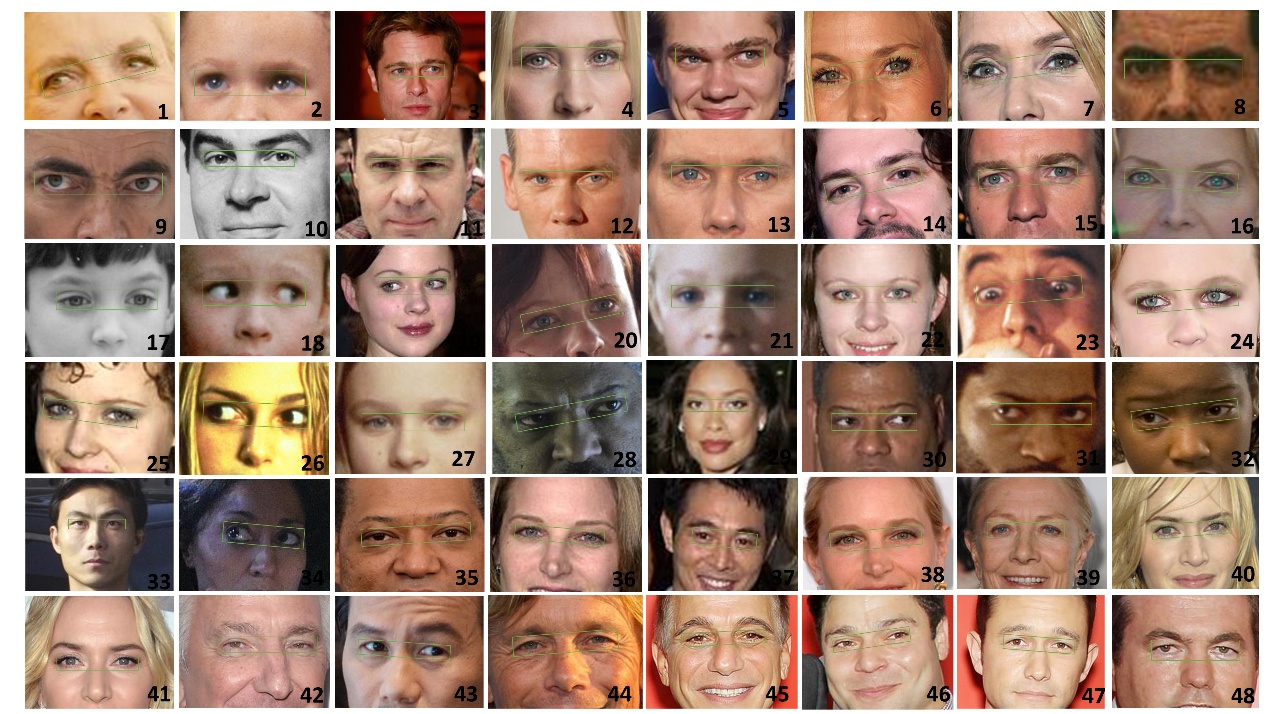


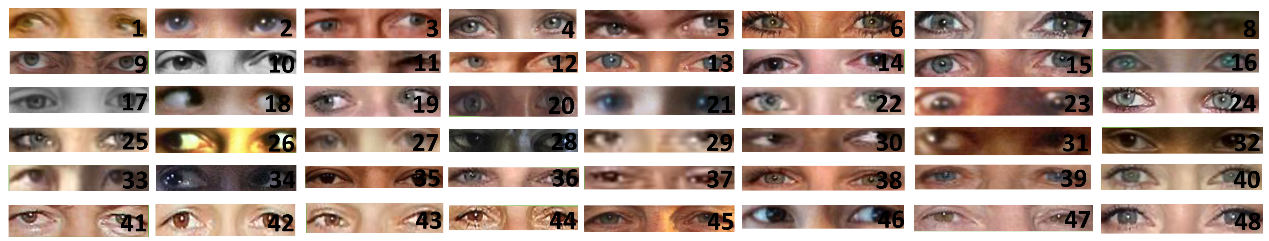


**Figure S4 Expert-Validated Eye Region Detection Across Diverse Strabismus and IMDB-WIKI Images**

Figure S4 illustrates the model's eye region detection performance across 96 images, comprising randomly selected 48 photographs from a Chinese strabismus patient dataset and 48 images from the IMDB-WIKI dataset, the largest publicly available face image collection (523,051 images) sourced from the web. These images span a diverse range of ages, skin tones, ethnicities, and distances, including obstructions such as fingers and cotton swabs. The cropping process focuses on retaining only the essential eye region, enhancing privacy for electronic health record systems and telemedicine applications. The model reduces distraction from non-essential facial areas, supporting advancements in artificial intelligence research for strabismus diagnostics based on gaze photos. Additionally, it uniquely identifies the eye region as a primary target and offers user-adjustable cropping boundaries, allowing expansion to include surrounding areas to cater to user preferences and enhance flexibility.

| **Table S1 The demographic composition of the study subjects and the key image attributes across the training and test datasets** | | |  |
| --- | --- | --- | --- |
| **Characteristics** | **Training set** | **Testing set** |  |
| Gender (Female/Male) | 327/273 | 24/24 |  |
| Images | 5400 | 432 |  |
| Age (years), median[range] | 7 [3, 37] | 6 [2, 74] |  |
| Ocular Alignment Angle (radians), median[range] | | 0 [-12.603, 5.737] | 0 [-5.713, 5.737] |
| Eye Region Bounding Box Area (percentage), median[range] | 0.134 [0.022, 0.305] | 0.127 [0.024, 0.249] |  |
| Exotropia (No), median[range](△) | 65,  -50 [-110, -10] | 16,  -40 [-120, -15] |  |
| Esotropia (No), median[range](△) | 22，  +40 [+10, +80] | 7,  +45 [+15, +140] |  |
| Vertical deviation (No),  L/R or R/L median[range](△) | 4，  10 [4, 20] | 3，  5 [4, 20] |  |
| Vertical deviation (No),  L/R or R/L median[range](△) | 4，  10 [4, 20] | 3，  5 [4, 20] |  |
|  |  |  |  |
